# Supplementary material for: We are What We Eat: Impact of Food from Short Supply Chain on Metabolic Syndrome
Source: J Clin Med. 2019 Nov 23;8(12):2061. doi: 10.3390/jcm8122061 (PMC6947359; doi:10.3390/jcm8122061)
Supplement: Supplementary file 1 [file jcm-08-02061-s001.pdf]

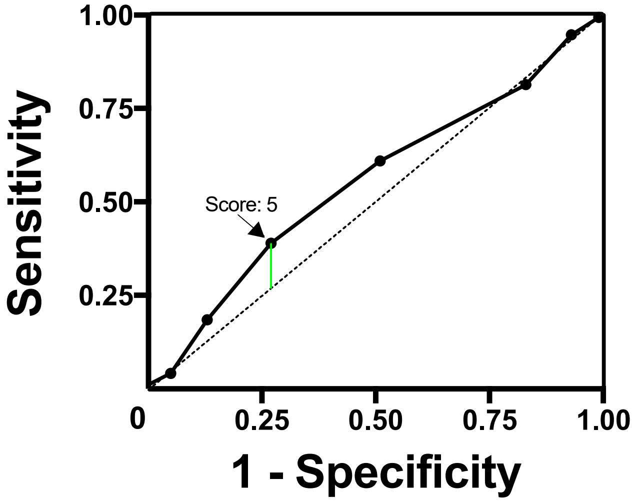

Supplementary Figure 1

Determination of the optimal cut off value in our questionnaire.

After having obtained the receiver operating characteristic (ROC) curve, we calculated the Youden's index; the 5 score displayed the highest Youden's J value (0.667), shown in green.
